# Antimicrobial activity and stability of the d-amino acid substituted derivatives of antimicrobial peptide *polybia*-MPI

**DOI:** 10.1186/s13568-016-0295-8

**Published:** 2016-11-29

**Authors:** Yanyan Zhao, Min Zhang, Shuai Qiu, Jiayi Wang, Jinxiu Peng, Ping Zhao, Ranran Zhu, Hailin Wang, Yuan Li, Kairong Wang, Wenjin Yan, Rui Wang

**Affiliations:** 1 Key Laboratory of Preclinical Study for New Drugs of Gansu Province, School of Basic Medical Sciences, Lanzhou University, 222 Tian Shui South Road, Lanzhou, 730000 People’s Republic of China; 2 The People’s Hospital in Gansu Province, 204 West Donggang Road, Lanzhou, 730000 People’s Republic of China

**Keywords:** Antimicrobial peptide, *Polybia*-MPI, Stability, d-Amino acid substitution

## Abstract

Antimicrobial peptide has the potential to be developed as new kind of antimicrobial agents with novel action mechanism. However, the susceptibility to protease is a drawback for potential peptides to be clinical used. d-amino acid substitution can be one way to increase the proteolytic stability of peptides. In the present study, we synthesized the d-lysines substituted analog (d-lys-MPI) and the d-enantiomer of *polybia*-MPI (D-MPI) to improve the proteolytic resistance of *polybia*-MPI. Our results showed that, the stability of its d-amino acid partially substituted analog d-lys-MPI was increased. However, it lost antimicrobial activity at the tested concentration with the loss of α-helix content. As shown in the CD spectra, after substitution, the spectra of D-MPI is symmetrical to MPI, indicated it turned into left hand α-helical conformation. Excitingly, the stability of D-MPI toward the tested protease was improved greatly. Notably, the antimicrobial activity of D-MPI was comparable to its L-counterpart MPI, even improved. In addition, the hemolytic activity of D-MPI was lowered. This also indicated that the action target of antimicrobial peptide *polybia*-MPI was not chiral specific. So, D-MPI may offer a therapeutic strategy to defend the infection of microbes, considering its stability to protease and relatively lower cytotoxicity to human erythrocytes.

## Introduction

Antimicrobial peptide (AMPs) is a component of innate defense system in most multicellular organisms, from humans to plants to insects. To date more than 2200 natural or synthetic AMPs have been reported through the antimicrobial peptide database (APD) (Silva et al. 2014). Antimicrobial peptides (AMPs) are generally composed of short sequences of 10–100 amino acids residues and highly amphipathic and membrane active (Reddy et al. 2004). AMPs have a broad spectrum of antibacterial, antifungal, antiviral, and anti-tumor activity at low concentrations. Unlike traditional antibiotics, antimicrobial peptides can regulate the host immune system and kill bacteria directly (Gottler and Ramamoorthy 2009). Furthermore, other biological activities of AMPs have also been described as following: neutralization of endotoxins, immune-modulating properties, chemokine-like activities, and induction of both angiogenesis and wound repair (Guaní-Guerra et al. 2010).

Although AMPs have broad spectrum of antimicrobial activity, the therapeutic applications of them still have many limitations, such as cytotoxicity, instability and high cost. Many antimicrobial peptides have cytotoxicity to mammalian cells at high concentrations, which may be due to membrane cracking mechanism (Marr et al. 2006). Moreover, AMPs could be degraded or removed by the endogenous protease or secreted by organism before arriving at the action sites. Therefore, their antimicrobial activities were greatly reduced or completely lost (Bowdish et al. 2005; Maisetta et al. 2008). The first commercial AMP was pexiganan acetate (MSI-78) which was an α-helical magainin variant peptide. MSI-78 has broad spectrum activity for treating foot ulcers and has developed into phase III clinical trials (Kang et al. 2012). BPI is a cationic protein from human neutrophil-derives which has antibacterial activity has just entered phase-III clinical trials (Ge et al. 1999; Hancock and Lehrer 1998). And up to now, no one have obtained US Food and Drug Administration (FDA) approval, except gramicidin for topical administrations (Steckbeck et al. 2014).

Recently, in order to reducing the toxicity and proteolytic degradation, many strategies were developed. It has been reported that the toxicity of AMPs can be reduced by modest sequence modifications (Kim et al. 2014). Strategies used to protect AMPs from protease degradation were mainly including cyclization, incorporation of non-proteinogenic amino acids, and sequence modification such as d-amino acids to replace the l-amino acids (Braunstein et al. 2004; Choi et al. 2013; Hamamoto et al. 2002; Lee and Lee 2008). Furthermore, several studies had reported d-amino acid substitution can improve the activity of AMPs or retain its activity, and more importantly can improve their stability.

*Polybia*-MPI, was a cationic peptide originally isolated from the venom of the social wasp *Polybia paulista* (Souza et al. 2005). Its primary sequence is IDWKKLLDAAKQIL-NH2 (1654.09 Da). We have demonstrated that *Polybia*-MPI displays potent antibacterial activity against both Gram-positive and Gram-negative bacteria. Furthermore, it was also found that *polybia*-MPI has potent antifungal activity and antitumor activity, and low toxicity to human red blood cells and normal fibroblasts (Wang et al. 2008, 2013). However, it is susceptive to the protease, which limits its clinical application. In the present study, its partial d-amino acid derivative d-lys-MPI (in which all l-lys was substituted by d-lys) and d-enantiomer (D-MPI) were synthesized by solid-phase peptide synthesis. Then their antimicrobial activity, hemolytic activity and stability were determined. Furthermore, the effect of such modification on the bioactivity, secondary structure and action mechanism was discussed.

## Materials and methods

### Synthesis of peptide

*Polybia*-MPI(I-D-W-K-K-L-L-D-A–A-K-Q-I-L-NH2 (1654.09 Da) and its analogues were synthesized by the standard Fmoc solid-phase peptide synthesis (Fields and Noble 1990; Wang et al. 2008). Each of synthesized peptide was purified to over 95% by reversed-phase high performance liquid chromatography (RP-HPLC) using a μ Bondapak C18 19- by 300-mm column. The flow rate was 8 ml/min and the gradient used was 5–95% solvent (CH3CN/H2O with 0.1% trifluoroacetic acid) over 60 min (Yan et al. 2012). The molecular weights were verified by matrix-assisted laser desorption ionization-time-of-flight mass spectrometry (ESI–MS). All the peptides were dissolved in deionized water and diluted by PBS before use.

### Antimicrobial assays

The minimal inhibitory concentration (MIC) of the peptides was measured in triplicate by the standard broth microdilution method (Peterson et al. 1980; Wang et al. 2012). In brief, bacteria/fungi were cultured overnight to mid-logarithmic-phase in Mueller–Hinton broth (MHB)/Sabouraud dextrose broth. The cultures were diluted to a final concentration of 2 × 10^4^ cfu/ml in fresh Mueller–Hinton broth (MHB)/Sabouraud dextrose broth. Each well of 96-well propylene microtitre plates was filled with 100 µl cell suspension and 100 μl peptide solution of twofold diluted serially. And the final concentrations of peptide mixtures ranged from 1 to 256 µM. Controls were done without peptides. After 18 h of incubation at 37 °C (bacteria)/35 °C (fungi), the minimal inhibitory concentration (MIC) of the peptides was evaluated by visible turbidity in each well.

The minimal bactericidal concentrations (MBCs) and minimum fungicidal concentration (MFCs) of the peptides was determined at the end of the incubation period by transferring 50 µl aliquots onto agar plates and incubated overnight at 37 °C/35 °C. The MBC/MFC was set as the lowest concentration of the number of CFUs that reduced at least 99.9%.

### Time-kill analysis

Strains was grown overnight with shaking to mid-logarithmic phases described above (Wang et al. 2015). The cultures containing 10^4^–10^5^ CFU/ml was incubated with different concentration of peptides which determined by MIC assay via the broth microdilution method. A no-peptide (control) tube was also included in each run. The cultures were incubated in a 37 °C/35 °C. shaking incubator. At different time intervals over the incubation period, samples from each tube were removed respectively and serially (tenfold) diluted, and 100 µl of the dilution were plated on agar plates. Plates were incubated at 37 °C/35 °C and determine the numbers of CFU after 24 h.

### Hemolytic activity

Fresh human blood was collected in heparinized-tube, diluted in cold PBS (pH 7.4) and centrifuged at 800*g* for 10 min to remove the serum and buffy coat. The erythrocytes were washed twice time and re-suspended in the same buffer to a final erythrocyte concentration of 8% for the hemolysis assay (Sharma and Sharma 2001). The RBC (red blood cell) suspension of 100 µl was transferred into a 96 well microtiter plate. Peptides were dissolved in PBS (pH 7.4) of different concentrations from 2 to 256 µM were incubated with 8% erythrocytes suspension for 60 min at 37 °C. Subsequently, the plate was centrifuged at 1200×*g* for 10 min. Then, aliquots of 100 µl supernatant were retransferred to an another 96-well plates and measured to monitor the release of hemoglobin by microplate reader (Bio-Rad 680) at 490 nm, which indicated RBC membrane damage. 100 µl RBC (red blood cell) suspension with 0.2% Triton-X 100 or PBS was used as positive and negative control, respectively. Hemolysis rate was determined as below:

$${\text{Hemolysis}} \,\,{\text{rate}} (\%) = ({\text{A}} - {\text{A}}2)/({\text {A}}1 - {\text{A}}2) \times 100\%$$

where A is the absorbance of indicator mixtures with different factors, A1 and A2 are the positive control, and the negative control, respectively (Kim et al. 2011).

### Circular dichroism spectroscopy analysis

The circular dichroism (CD) spectra of peptides were measured on an Olis DSM 1000 CD spectrophotometer (USA) in spectra between 195 and 240 nm under nitrogen flush in 1 mm path length at room temperature. The scanning speed was 100 nm/min, 2 s response time and 1.0 nm spectral band width. The measurements were performed with a peptide concentration of 50 mM. The spectra were measured in 50% TFE (v/v) and 10 mM PBS (pH 7.4), and the average of four scans was taken.

### Assessment of stability

#### Viability assay

Viability assay different concentration of trypsin (from 0.0002 to 2 mg/ml) incubated with peptides for 1 or 6 h at 37 °C, and the enzymatic activity was terminated by additional heat inactivation at 60 °C for 20 min. Then the trypsin treated peptides were incubated with *Escherichia coli* (ATCC25922) at the concentration of MIC in 96-wells microtiter plate overnight at 37 °C (Choi et al. 2013). The growth inhibitory effect was determined by measuring the absorbance at 600 nm. Then we took 100 μl samples of 6 h from each well with the concentration of 0.2 mg/ml and plated onto MH broth ager after dilution. After overnight incubation at 37 °C, photos of plates were photographed and the colonies on the plate were counted.

#### Radial diffusion assay

The enzymatic stability of *Polybia*-MPI and its analogues was evaluated by a modification of the sensitive radial diffusion assay. The bacteria were cultured until the optical density at 600 nm (OD600) of an aliquot reached 0.5. One milliliter of the bacteria suspension was added to 100 ml of previously autoclaved, warm Mueller–Hinton agar (MHA)/Sabouraud dextrose agar. The medium were poured into layer of a 5 mm deep. Wells were performed with a sterile plastic bore of a 3-mm-diameter gel punch. Following the addition of 20 µl pre-incubated mixture of peptides and different concentrations of trypsin or chymotrypsin for 4 h into wells. The plates were incubated at 37 °C for 18 to 24 h. An equal volume of 20 µl mixture had no trypsin or chymotrypsin was used as the control. The size of the clear zones surrounding each well as indicators of the enzymatic stability of *Polybia*-MPI and its analogues.

### RP-HPLC analysis

The stability of *polybia*-MPI and its analogues was assayed with trypsin or chymotrypsin in a volume ratio of 1:15 (peptide:trypsin) (Choi et al. 2013; Hamamoto et al. 2002; Molhoek et al. 2011). Peptides at the concentration of 10 mM were incubated with trypsin or chymotrypsin (0.2 mg/ml) at 37 °C. 40 µl samples of the incubations were taken for the time of 0 and 360 min, respectively. Then, the samples were mixed with 80 µl of 15% trifluoroacetic acid (TFA) and 80 µl acetonitrile which incubated at 4 °C for at least 15 min to precipitate proteins. The supernatant was collected of each sample after centrifugation at 13,000 rpm for 10 min and were detected with reverse phase high performance liquid chromatography (RP-HPLC) analysis.

### Confocal laser scanning microscopy

*Candida glabrata* cells were cultured overnight to mid-logarithmic-phase in Sabouraud dextrose broth. After centrifugation and suspension, the cells were incubated with *polybia*-MPI and its analogues at final concentration of 5× MIC for 2 h at room temperature. Then, PI (50 µg/ml) was added and was further incubated for 5 min in the dark (Wang et al. 2012).

## Results

### Antibacterial and bactericidal activity

**Fig. 1:**
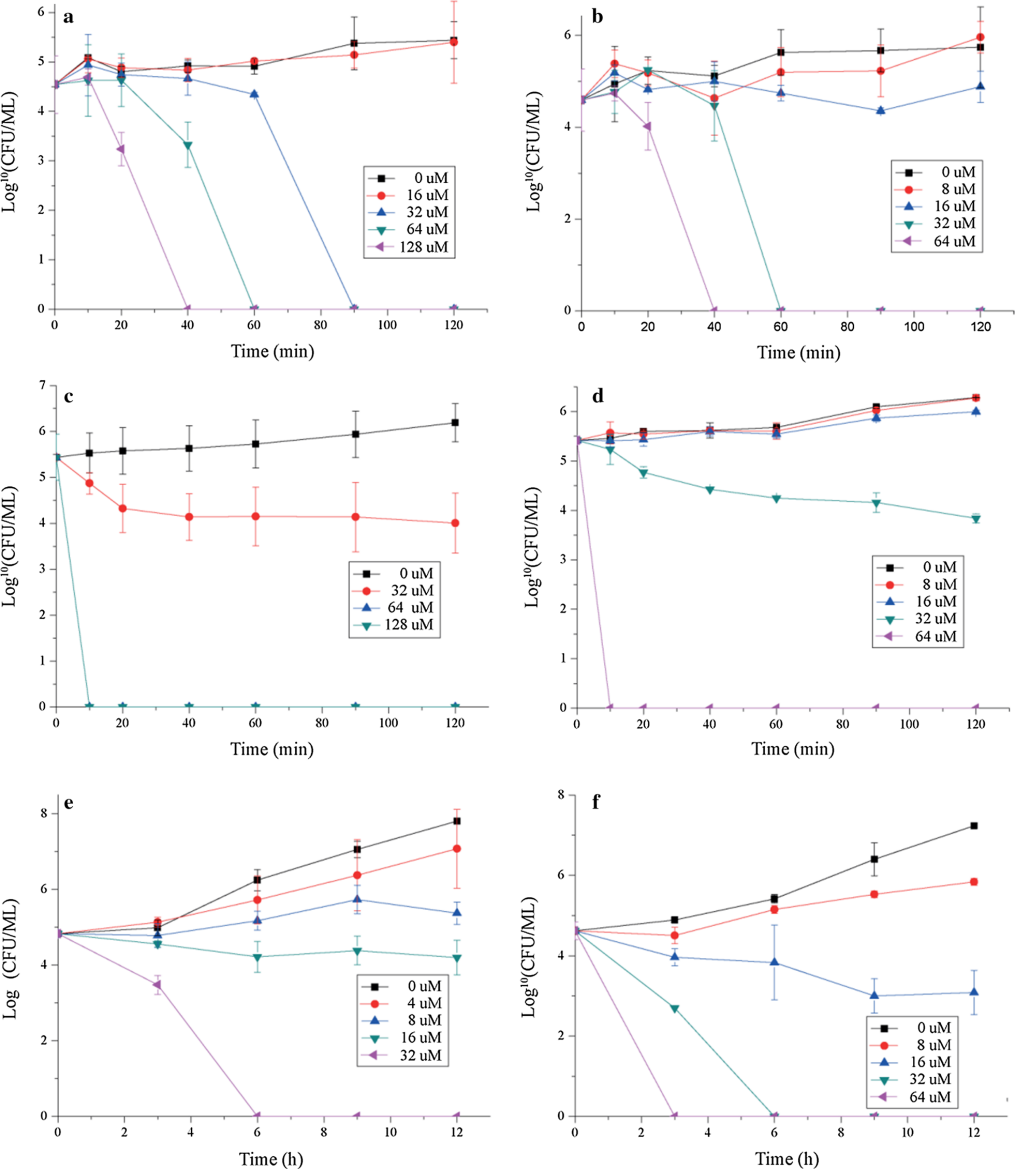
Time-killing kinetics curves for *polybia*-MPI and D-MPI against *E. coli* (**a** and **b**), *S. aureus* (**c** and **d**) and *C. glabrata* (**e** and **f**). Standardized cells suspensions were incubated with a final concentration of peptides of 1/2× MIC, 1× MIC, 2× MIC, 4× MIC. At different time intervals, samples were taken respectively from each tube and serially diluted. Then 100 µl of the dilution were plated for colony counts. Controls were without peptide. Each data point represents mean result ± standard deviation (*error bars*)

The antimicrobial activity of d-lys-MPI and D-MPI against bacteria and fungi were determined. As shown in Table 1, D-MPI could potently inhibit the growth of all tested bacterial and fungal strains with MIC values almost in the range of 8–64 µM. However, d-lys-MPI was inactive against bacteria and much less active against fungal strains at the tested concentration. Compared with *polybia*-MPI, the inhibitory effects of D-MPI was improved more or less, which also could be seen in the bactericidal or fungicidal activity (Table 2). As shown in Fig. 1, the improvement of bactericidal or fungicidal activity also could be reflected in the time-killing curves of D-MPI against the tested strains.

**Table 1:** MIC values of *polybia*-MPI, D-MPI and d-lys-MPI against the tested Bacteria cells and Candida cells

Organism	MIC(µM)		
	*Polybia*-MPI	D-MPI	d-lys-MPI
Bacteria			
*E. coli (ATCC 2592)*	32	16	>256
*E. coli (ML*-*35) (ATCC 43827)*	32	32	>256
*Klebsiella influenza (ATCC 700603)*	64	64	>256
*Pseudomonas aeruginosa (ATCC 27853)*	128	64	>256
*sakazakii (ATCC 29544)*	64	32	>256
*Staphylococcus aureus (ATCC 29213)*	32	16	>256
*Bacillus subtilis (ATCC 23857)*	8	8	>256
*Staphylococcus epidermidis (ATCC 12228)*	32	16	256
Fungi			
*Candida glabrata ATCC (ATCC 2001)*	8	16	128
*Candida albicans ATCC (ATCC 14053)*	16	16	256
*Candida parapsilosis (ATCC 22019)*	64	32	>256
*Candida tropicalis (ATCC 750)*	8	16	64
*Candida krusei (ATCC 6258)*	8	8	128

**Table 2:** MBC/MFC values of *polybia*-MPI, D-MPI and d-lys-MPI against the tested Bacteria cells and Candida cells

Organism	MBC/MFC(µM)		
	*Polybia*-MPI	D-MPI	d-lys-MPI
Bacteria			
*E. coli (ATCC2592)*	32	16	>256
*E. coli (ML*-*35) (ATCC43827)*	256	64	>256
*Klebsiella influenza (ATCC 700603)*	256	128	>256
*Pseudomonas aeruginosa (ATCC27853)*	>256	128	>256
*sakazakii (ATCC 29544)*	64	64	>256
*Staphylococcus aureus (ATCC29213)*	128	64	>256
*Bacillus subtilis (ATCC23857)*	8	8	>256
*Staphylococcus epidermidis (ATCC 12228)*	32	128	>256
Fungi			
*C. glabrata (ATCC 2001)*	32	32	256
*C. albicans (ATCC 14053)*	16	16	>256
*C. parapsilosis (ATCC 22019)*	128	32	>256
*C.tropicalis (ATCC 750)*	32	16	128
*C. krusei (ATCC 6258)*	16	8	256

### Hemolytic activity

**Fig. 2:**
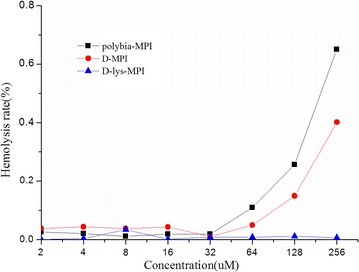
The hemolytic activity of *polybia*-MPI and synthetic D-MPI and d-lys-MPI against human erythrocytes were determined. Fresh human blood was collected and centrifuged to remove the serum and buffy coat, and re-suspended to a final erythrocyte concentration of 8% erythrocytes suspension. Then, peptides of different concentrations from 2 to 256 μM were incubated with erythrocytes suspension for 60 min. Subsequently, the erythrocytes suspension was centrifuged and measured to monitor the release of hemoglobin at 490 nm

The hemolytic activity of *polybia*-MPI, D-lys-MPI and D-MPI against human erythrocytes was determined with the concentration ranging from 1 to 256 µM. As indicated in Fig. 2, no significant hemolytic activity was found under the concentration of 100 μM. Compared to its L-counterpart *polybia*-MPI, the hemolytic activity of D-MPI was decreased after the d-amino acid substitution. After substitution of l-lysine by d-lysine in the peptide sequence, the hemolytic activity also was abrogated.

### Circular dichroism spectroscopy analysis

**Fig. 3:**
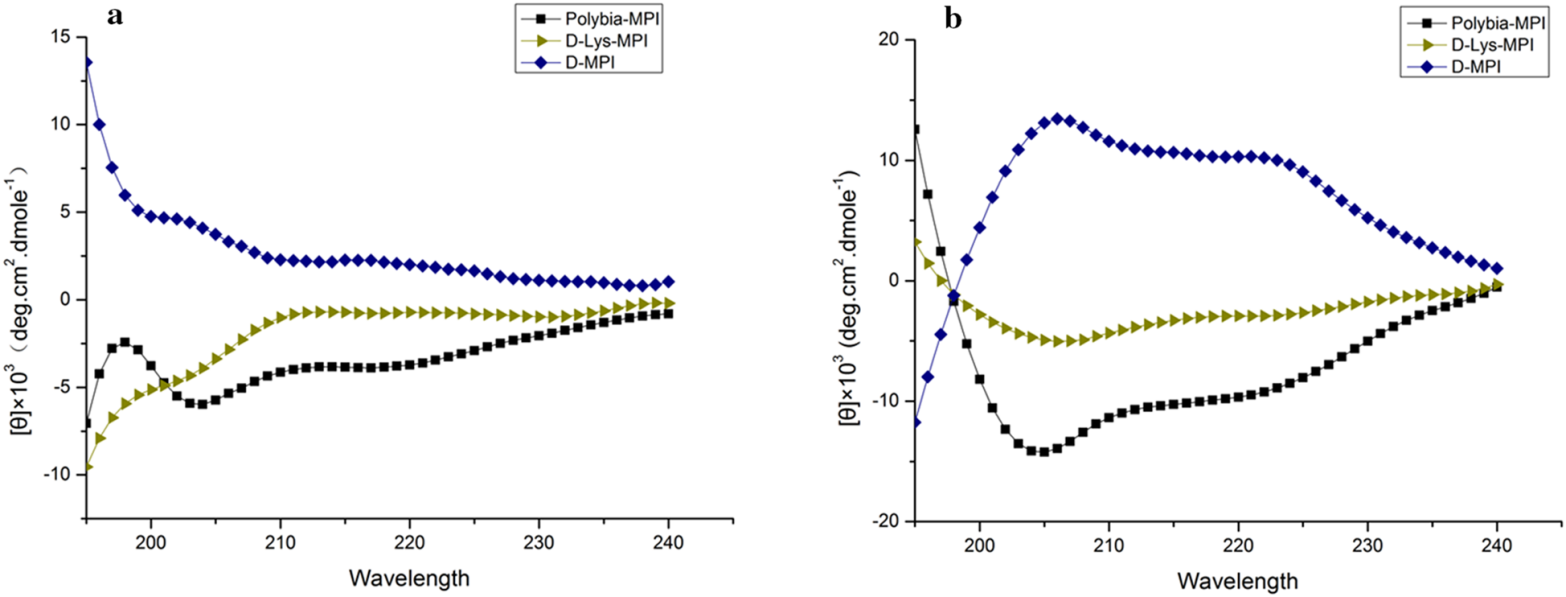
Circular dichroism spectra analysis of *polybia*-MPI and its analogues. **a** sodium phosphate buffer. **b** Membrane-mimicking environment (50% TFE)

The secondary structure of these peptides were determined in 10 mM sodiumphosphate buffer (PBS, pH 7.4) and a membrane-mimicking environment (50% 2,2,2-trifluoroethanol) by circular dichroism spectroscopy. As shown in Fig. 3, all the peptides displayed a unordered structure in PBS. In the membrane-mimicking environment, D-MPI showed a negative band at 195 nm, and two positive bands near 208 and 222 nm, which was mirror symmetrical to its L-counterpart *polybia*-MPI. D-MPI takes a left hand α-helical conformation in the membrane-mimicking environment. However, the d-lys-MPI showed a decreased α-helical conformation after the substitution of l-lysine with d-lysine for there is a quite slightly tendency of negative bands around 208 and 222 nm.

### Stability of the peptides

#### Viability assay

**Fig. 4:**
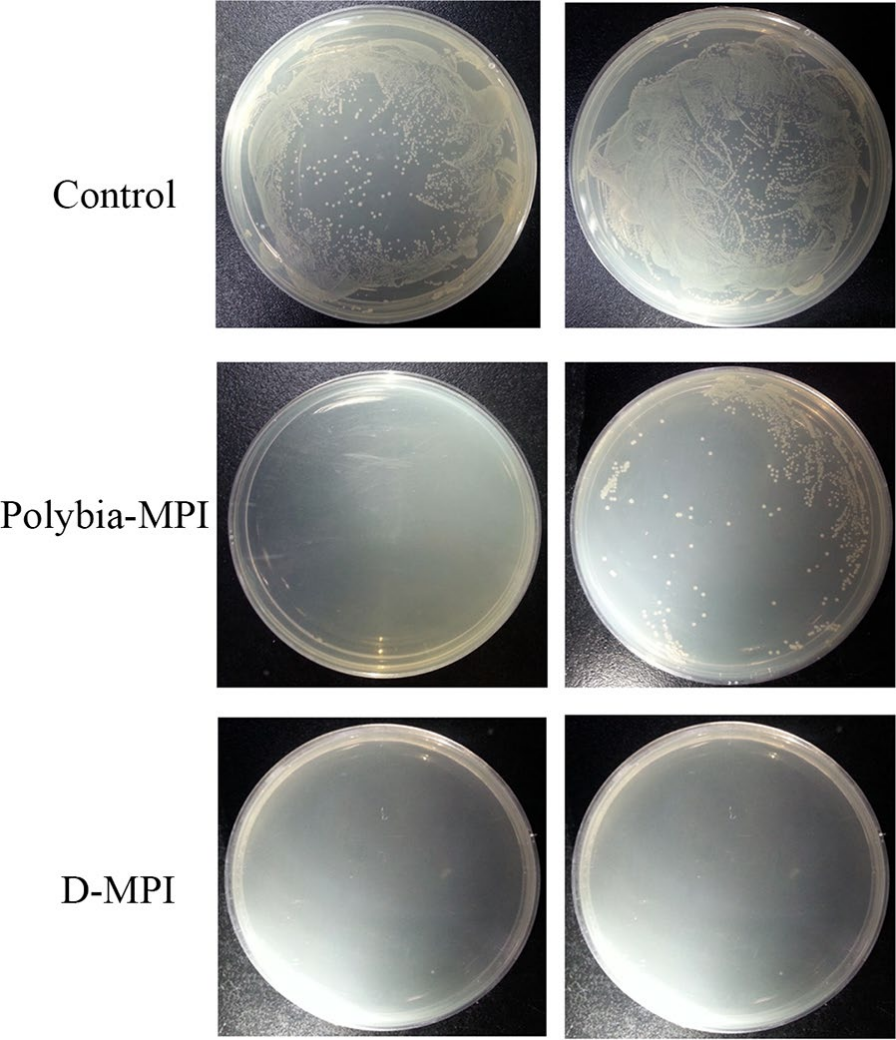
The effect of protease on the bactericidal activity of *polybia*-MPI and D-MPI. Peptides were incubated with the same volume of PBS (*Left*) or trypsin (*Right*) at 37 °C for 4 h. The enzyme was heat inactivated at 60 °C for 20 min, and *E. coli* suspension was added and co-incubated for 3 h. Then, the mixtures were plated onto MH broth ager and incubated for 18–24 h. Pictures of each plate were photographed

**Fig. 5:**
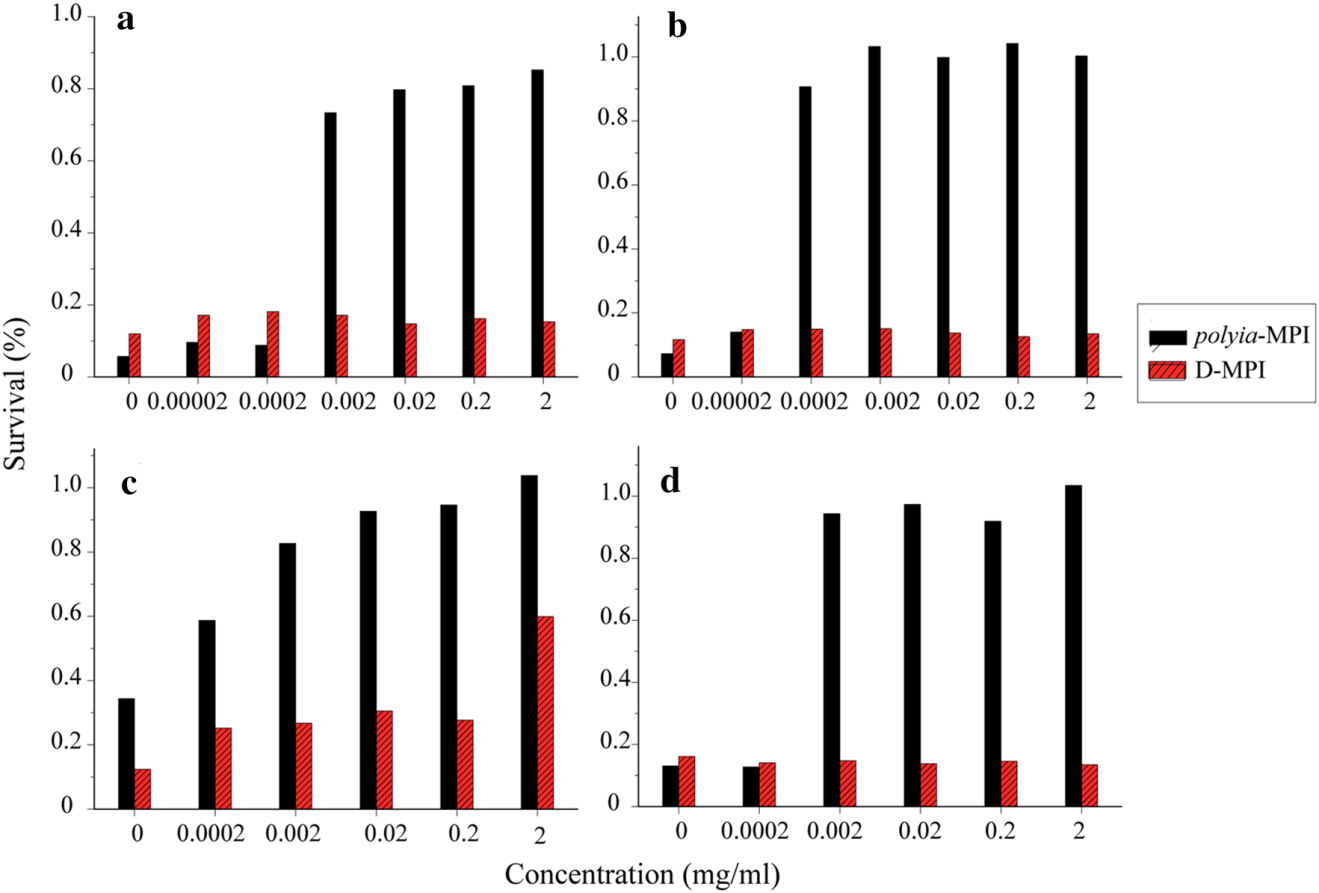
Inhibition effect of protease on the antimicrobial activity of *polybia*-MPI and D-MPI. The proteases used were trypsin (**a** and **b**) and chymotrypsin (**c** and **d**). Peptides with proteases of different concentration which ranged from 0.0002 to 2 mg/ml (tenfold) were incubated for 1 h (*left*) or 6 h (*right*) at 37 °C, respectively. Then tested for its antimicrobial activity against *E. coli* by determine the OD values at 600 nm

**Fig. 6:**
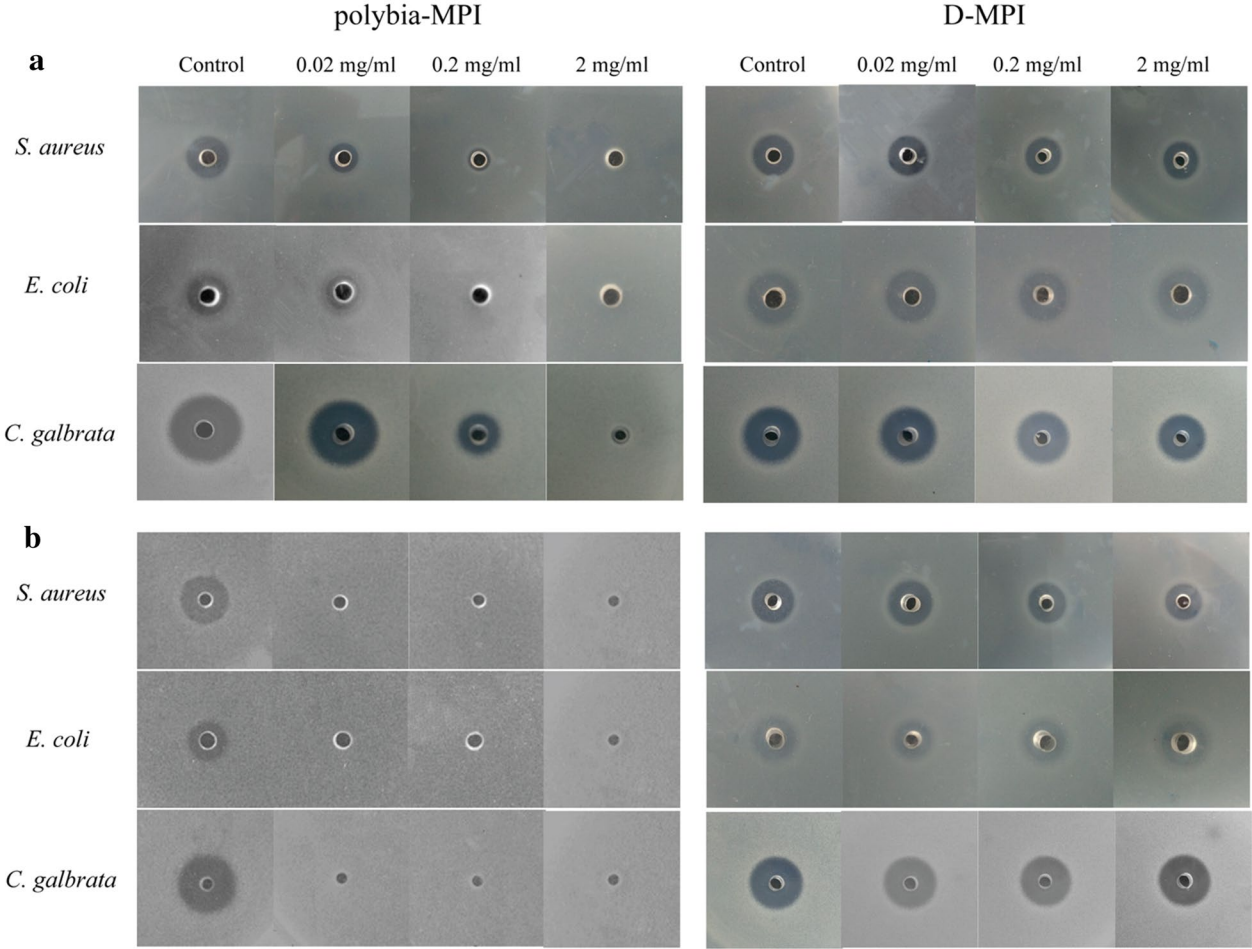
The viability of bacteria/fungi after incubation with peptides in the presence/absence of enzyme. Each peptide was pre-incubated with different concentrations of chymotrypsin (**a**) or trypsin (**b**) at 37 °C for 4 h. Then the samples were heat inactivated and poured into wells of plates. The plates were incubated at 37 °C for 18–24 h. Controls were without trypsin or chymotrypsin

One of the major limitation of AMPs for clinical use is that AMPs could be easily degraded by the endogenous proteases. To test the stability of *polybia*-MPI and D-MPI, first 1 mg/l of the peptides were incubated with trypsin and chymotrypsin for 4 h for degradation. After the enzyme was heat inactivated at 60 °C for 15 min, *E. coli* was added and co-incubated for 3 h. Then, the treated bacteria were coated to plate and incubated at 37 °C for 24 h. As shown in Fig. 4, there are no colony grown on the trypsin-D-MPI-treated plate, while there are many colonies grown on the trypsin-MPI-treated plate. D-MPI showed significant protease resistance against trypsin. To further examine the susceptibility of peptides to protease, peptides was incubated with the different concentration of trypsin (a and b) and chymotrypsin (c and d) (ranged from 0.0002 to 2 mg/ml) for 1 and 6 h respectively, then the relative survival rate of *E. coli* were determined. As shown in Fig. 5, trypsin and chymotrypsin could totally inhibit the antimicrobial activity of *polybia*-MPI at the concentration of 0.002 mg/ml after incubation 1 h, while D-MPI retained its antimicrobial activity after incubation with 2 mg/ml of trypsin and chymotrypsin for 6 h. This result was further confirmed by radical diffusion assay. As shown in Fig. 6, the inhibition zone diameters in *polybia*-MPI treated group were decreased even disappeared after incubation with 0.02–2 mg/ml of trypsin and chymotrypsin, respectively. However, in the D-MPI treated group the inhibition zone diameters was almost the same as control after the incubation with 2 mg/ml of tested proteases.

#### RP-HPLC analysis

**Fig. 7:**
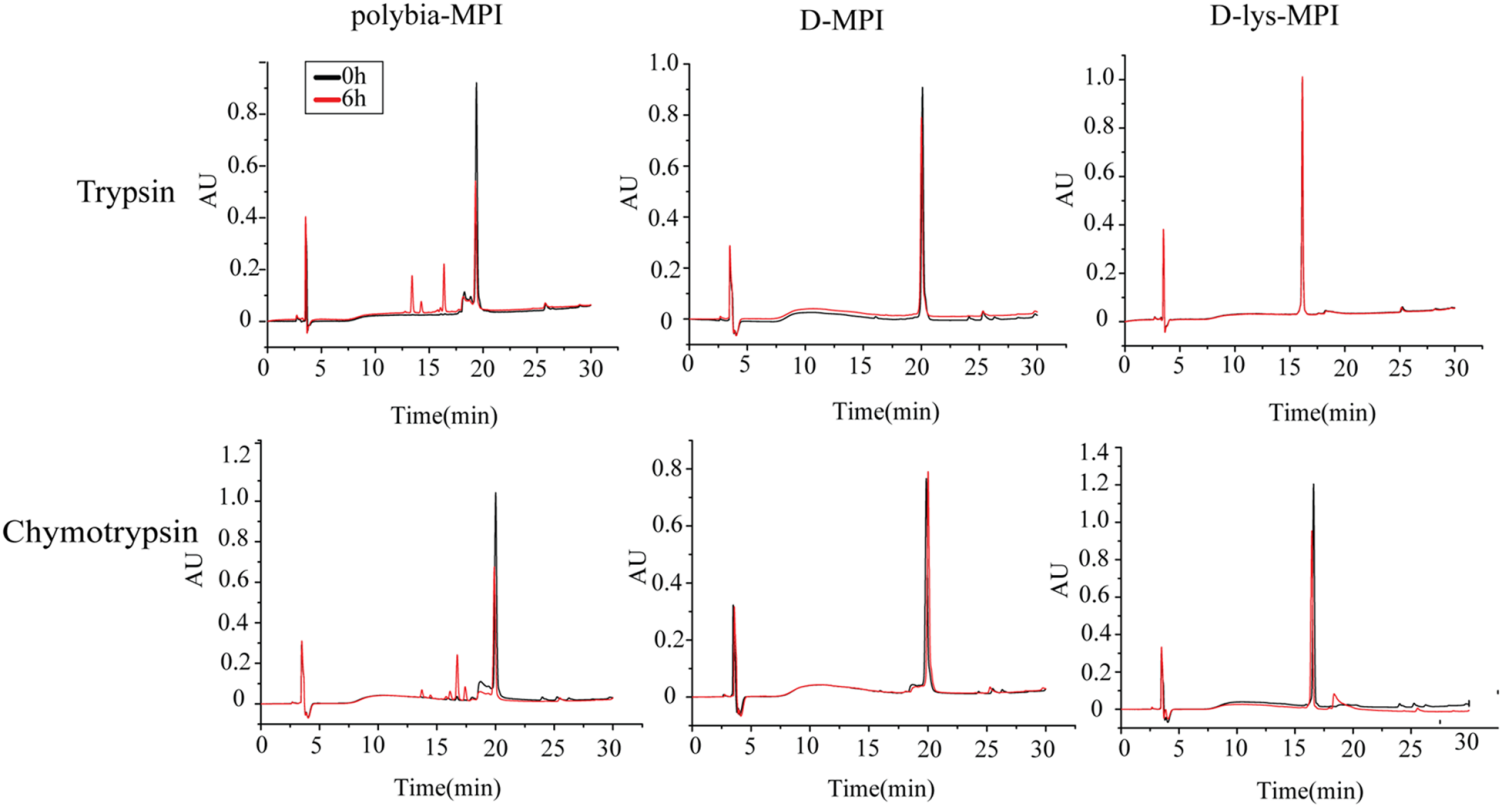
HPLC analysis of the susceptibility of *polybia*-MPI, D-MPI and D-lys-MPI to trypsin and chymotrypsin. Peptides at the concentration of 10 mM were incubated with trypsin or chymotrypsin at 37 °C. At the determined time interval, samples were taken and mixed with trifluoroacetic acid (TFA) and acetonitrile to precipitate proteins, respectively. Then the samples were detected with reverse phase high performance liquid chromatography (RP-HPLC) analysis

In the present study, HPLC was used to determine the stability of *polybia*-MPI, d-lys-MPI and D-MPI to trypsin, chymotrypsin and proteases in human serum. When comparing the HPLC profile of untreated peptides to the HPLC profile of trypsin and chymotrypsin treated peptides, it was found that *polybia*-MPI was susceptible to all the tested enzymes and serum, while its partially d-lysine substituted analogue and fully d-amino acid substituted were not or only partly degraded after treatment (Fig. 7).

### Peptides induced cell membrane permeabilization

**Fig. 8:**
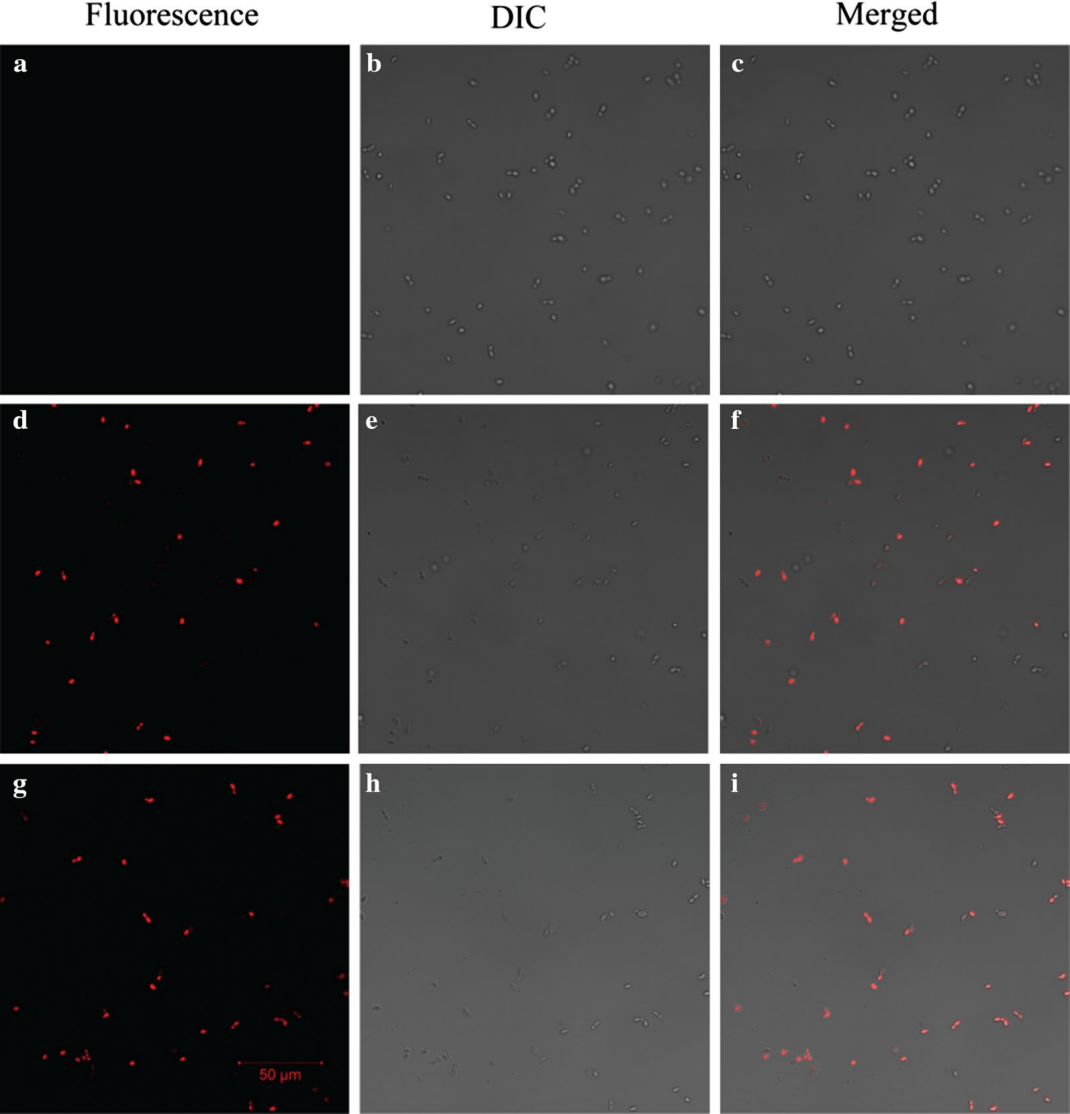
Detection of peptides-induced PI uptake by LSCM. Mid-logarithmic-phase *C. glabrata* cells were incubated with 0 µM or 5× MIC peptides for 2 h. Then, PI (50 µg/ml) was added and was further incubated for 10 min in the dark. PI uptake was detected by LSCM. The control was done without peptide. Control (**a**–**c**); *polybia*-MPI (**d**–**f**); D-MPI (**g**–**i**). *Bars*, 50 µm

To determine the effect of D-MPI on the integrity of the fungal cell membrane, PI uptake assay was employed in the present study. PI was a kind of DNA intercalating fluorescent dye, which could only pass through the damaged cell membranes and combined with DNA to form a PI-DNA complex. As shown in Fig. 8, there is no fluorescence in the control group (A–C), while the D-MPI treated group (G–I) showed fluorescence as its L-counterpart treat group (D–F), indicating that D-MPI also could disrupt the integrity of the cell membrane.

## Discussion

Since the golden age of antibiotics, antibiotics had truly become the “panacea” in the field of medicine and were being used to treat even the most common infections. However, spawned a series of multi-drug resistant bacteria with the indiscriminate use and abuse of antibiotics,traditional antibiotics have been unable to meet the needs of clinical medicine (Wang et al. 2016). Therefore, the urgent need for new antibiotics has stimulated the rapid development of antimicrobial peptides. Most antimicrobial peptides are presented with a positive charge at neutral PH and have an amphiphilic topology, so they can combined with a negatively charged phospholipid head, and ultimately damage membrane structure through interactions with cytoplasmic membrane (Wang et al. 2013). Due to these special properties, antimicrobial peptides are therefore considered to be a potential alternative to traditional antibiotics. Although AMPs have broad spectrum antimicrobial activity in vitro, there are many obstacles in clinical application, such as cell toxicity, instability and high cost (Kim et al. 2014). To solve these problems, many methods have been reported. For instance, substitution Trp and Phe residues with less hydrophobic amino acids can decreases the toxicity of AMPs (Kim et al. 2014). Introduction of non-natural amino acids (mainly d-form amino acids), cyclization or modification the terminal regions by acetylation or amidation can improve the stability of peptides (Wei and Bobek 2005; Kim et al. 2014).

In nature, almost all of the proteins and peptides were composed of l-amino acid. Different enzyme could recognize specific peptide bond formed by l-amino acid and break it (Chen et al. 2016). However, enzyme could not break the peptide bond formed by d-amino acid for the difference of the spatial configuration. Actually, there is no difference between l-amino acid and its D-counterpart in the chemical and physical property. So, d-amino acid substitution almost has no influence on the chemical and physical property of the peptides except the configuration. As the influence of d-amino acid substitution on the biological activity of peptides, it is depend on that if the action mechanism related to the recognition of chiral targets (Braunstein et al. 2004). In the present study, when only the three Lysines in the sequence of *polybia*-MPI were substituted by their D-counterparts, the secondary structure was changed compared to *polybia*-MPI and the peptide almost lost all the biological activity in the tested concentration. However, when all the l-amino acids in the sequence of *polybia*-MPI were substituted by d-amino acids, the configuration was reversed. The D-enantiomer of *polybia*-MPI was turned into left hand α-helical conformation. More importantly, all d-amino acid substitution didn’t affect its biological activity. D-MPI has the same antimicrobial activity even improved activity as *polybia*-MPI.

Furthermore, the action mechanism of D-MPI also was analyzed in the present study. The action mode of AMPs has been investigated in many studies. Many AMPs were found that they could bind with the membrane of microbes and then disrupt the integrity of cell membrane (Arnusch et al. 2012). There are mainly three models, the barrel-stave model, the toroidal pores model, and the carpet model (Chih et al. 2015), which are widely accepted to describe the membrane-active action mode. Membrane active action mode of AMPs was believed that it is difficult for microbes to develop resistance toward AMPs (Hancock and Sahl 2006). The L-counterpart of D-MPI mainly exerts its antitumor, antibacterial and antifungal activity through the disruption of the integrity of cell membrane (Wang et al. 2008, 2009, 2013, 2014). As expected, it may also exert its antimicrobial activity through disrupting the integrity of cell membrane and lead to the death of cells. This kind of cell membrane lysis action mechanism always was not dependent on the chiral recognition, which support that D-MPI retained the activity of its l-counterpart. Interestingly, all d-amino acid substitution also decreased the hemolytic activity of *polybia*-MPI.

In conclusion, our results showed that the fully substitution of all the amino acid of *polybia*-MPI with d-amino acid could protect the peptide from the degradation of the tested proteases. This modification turned right hand α-helical conformation of *polybia*-MPI to left hand conformation and retained or improved the antimicrobial activity of *polybia*-MPI. Although the partially d-lysine substitution could improve the stability of *polybia*-MPI toward the tested protease, the secondary conformation was changed after modification and its antimicrobial activity decreased greatly. So, D-MPI may offer a new strategy and will be an excellent alternative to conventional antibiotics to defend the resistant bacteria.
